# Development and Application of Gas Production Measurement System of Coal-Rock under Temperature–Pressure Coupling

**DOI:** 10.3390/s22186776

**Published:** 2022-09-07

**Authors:** Chunde Ma, Zihe Wang, Jiaqing Xu, Guanshuang Tan, Zhihai Lv, Quanqi Zhu

**Affiliations:** 1 School of Resources and Safety Engineering, Central South University, Changsha 410083, China; 2 Institute of Mechanics for Engineering Materials, Advanced Research Center, Central South University, Changsha 410083, China

**Keywords:** coal-rock, coalbed methane, MTS-815, gas production, temperature–pressure coupling

## Abstract

In this study, a measurement system for gas generation of coal-rock under temperature–pressure coupling was developed by adding gas extraction, collection, and flow-monitoring devices to the original stainless-steel liquid seepage pipeline of an MTS-815 rock triaxial testing machine, which can be used to study the production mechanism of coalbed methane in a real geological environment. The system has the functions of axial loading, confining pressure loading, continuous heating, gas gathering, etc., and has the advantages of good air tightness, high accuracy and stability, long-term loading and heating, and controllable single variables. The preliminary test for the gas production of anthracite in the Shaanxi Formation of the Qinshui Basin under temperature–pressure coupling was carried out by the developed test system. The results show that the test system can provide accurate and effective measurement means for the study of gas production by coal-rock deformation and is expected to provide effective help for the control and exploitation of coalbed methane.

## 1. Introduction

In view of the environmental pollution and carbon emission caused by traditional fossil resources, the development and utilization of new energy resources led by natural gas have become the focus of development in the energy industry and mining field in the 21st century [1]. On one hand, coalbed methane (CBM) is a methane-based gaseous geological body produced by chemical and tectonic processes of coal and belongs to unconventional natural gas. Its calorific value is 2–5 times higher than that of traditional coal resources, and almost no exhaust gas is produced after combustion [2,3], so the CBM is an efficient and clean energy resource. On the other hand, as a gas resource associated and symbiotic with coal, CBM often leads to a series of serious coal mine disasters such as gas poisoning, spontaneous combustion, and gas explosions in the process of coal mining [4,5,6,7,8,9]. In addition, if CBM is directly discharged into the atmosphere, it will cause great damage to the ecological environment because its greenhouse effect is approximately 21 times that of carbon dioxide. Therefore, extracting CBM in a coalbed before coal mining can not only greatly reduce coal mine disasters and environmental damage but also obtain a large amount of clean energy resources and generate considerable economic benefits.

Many scholars have carried out research on the disasters and efficiency of CBM exploitation [10,11,12,13,14,15,16,17]. However, since the natural form of CBM molecules is mainly adsorbed on the surface of coal matrix particles or dissociated in the pores of coal-rock mass, or even dissolved in coalbed water, it is difficult to achieve direct and efficient extraction. In recent years, some scholars have tried to solve the CBM production problem from the perspective of the geophysical characteristics of CBM [18,19,20,21,22]. However, due to the lack of accurate quantitative characterization methods and detection techniques for many key physical parameters such as gas molecular adsorption, gas content, and the gas yield rate in coal-rock under pressure and temperature coupling [23,24,25,26], there is still a lack of clear and reliable understanding of the gas release mechanism of coal-rock in complex geomechanical environments, which in turn makes it difficult to put forward safe and efficient technical methods for CBM exploitation or disaster prevention.

For this reason, many scholars have attempted to reveal the mechanism of coal-rock gas production [27,28,29,30,31,32,33,34,35], and the research was mainly focused on coal metamorphism gas production, microbial gas production, biochemical gas production, and gas production by coal’s mechanochemical deformation. They verified the metamorphism of coal-rock and the effect of gas generation from the macroscopic and microscopic structure of coal-rock, and explored the gas production mechanism by using experimental methods. However, due to the influence of the traditional theory of gas production by coal pyrolysis and high-temperature pyrolysis, most experimental studies imposed high-temperature and high-pressure environments on coal-rock, resulting in the phenomenon of gas production by coal-rock deformation being generally ignored [31,32,33,34,35,36]. Furthermore, there were several major problems with previous experimental methods. For example, the actual occurrence environment of coal-rock is under the conditions of three-dimensional in-situ stress and certain geothermal conditions, while many studies were carried out using a simple uniaxial compression device, which could not fully simulate the real CBM exploitation environment [30]. Although the confining pressure was considered in some tests, the self-made experimental device generally had the problem in which the kettle body and the coal-rock specimen could not be completely fitted, and the insufficient air tightness of the experimental device would lead to low accuracy of the experimental results [31,32]. In addition, the temperature conditions were considered in some tests, but the experimental temperature (350~700 °C) used was much higher than the initial temperature of pyrolysis or even the cracking of coal specimens, which made it impossible to identify and analyze the gas generated only by coal-rock deformation [33].

In view of this, based on the MTS-815 rock triaxial test machine, a new measurement system that can simulate the gas production of coal-rock under temperature–pressure coupling is developed, and the gas production by coal-rock deformation under temperature–pressure coupling is preliminarily tested using this system. This study can provide the technology and method for revealing the deformation and gas production mechanism of coal-rock in a real geological environment.

## 2. Design Ideas

In this study, a measurement system that can accurately measure the gas production of coal-rock under temperature–pressure coupling was designed and developed based on the MTS-815 rock triaxial test machine. The main design ideas are as follows: (1) Using the accurate axial pressure and confining pressure loading function of the MTS-815 test machine to simulate the real three-dimensional in situ stress conditions borne by coal-rock at different buried depths, and the axial and circumferential deformation of the coal-rock specimen during the loading are recorded by extensometers; (2) using the heating function of the MTS-815 test machine to simulate the geothermal gradient environment of coal-rock; (3) using the sealed pipeline with the liquid seepage function in the triaxial chamber of the MTS-815 test machine, and gas extraction devices such as a vacuum air pump, flowmeter, gas collection bag, etc., outside the triaxial chamber, to collect and measure all the gases generated by the coal-rock specimen under the temperature–pressure coupling; and (4) using high-precision gas chromatography–mass spectrometry (GC-MS) to analyze the components of the collected gases. In this way, the gas composition and gas yield generated by the coal-rock under different temperature and pressure combinations can be known, and thus, the gas production mechanism can be revealed. The development of this measurement system is expected to provide a reference and optimize the operation and management for actual coal mining operations and CBM exploitation processes, improve the extraction and utilization efficiency of new energy sources such as CBM, and prevent coal mine gas disasters.

## 3. Establishment of the Test System

**Figure 1:**
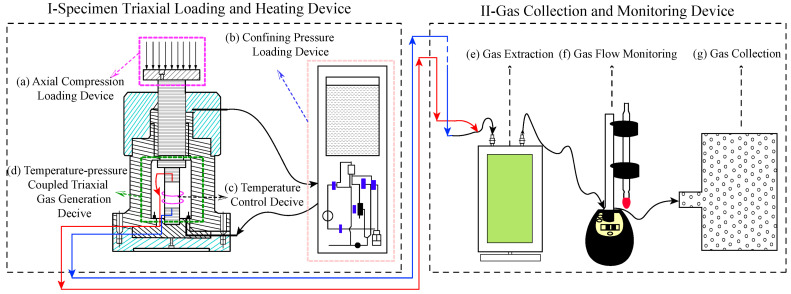
Schematic diagram of gas production measurement system of coal-rock under temperature–pressure coupling.

Figure 1 shows the schematic diagram of the gas production measurement system of coal-rock under temperature–pressure coupling based on the MTS-815 triaxial test machine. The test system mainly includes two parts: The specimen triaxial loading and heating device and the gas collection and monitoring device. The specimen triaxial loading and heating device is based on the MTS-815 test machine, which takes charge of axial and confining pressure loading, heating, and constant temperature control on the specimen, so as to simulate the complex geological environments of coal-rock. The functions of the gas collection and monitoring device include gas flow monitoring, gas extraction, and the collection of coal-rock gas. The details of each test device and method are as follows.

### 3.1. Specimen Triaxial Loading and Heating Device

The specimen triaxial loading and heating device includes a temperature–pressure-coupled triaxial gas generation device and a confining pressure loading device. The specimen is subjected to axial loading and continuous heating by the temperature–pressure-coupled triaxial gas generation device, and the confining pressure is applied radially on the specimen by the confining pressure loading device. In order to prevent the accuracy of experimental results from being affected by the pyrolysis reaction of gas components in coal-rock at a high temperature, the test temperature is controlled below 200 °C, and the influence of temperature on the reaction rate is compensated by prolonging the loading time.

**Figure 2:**
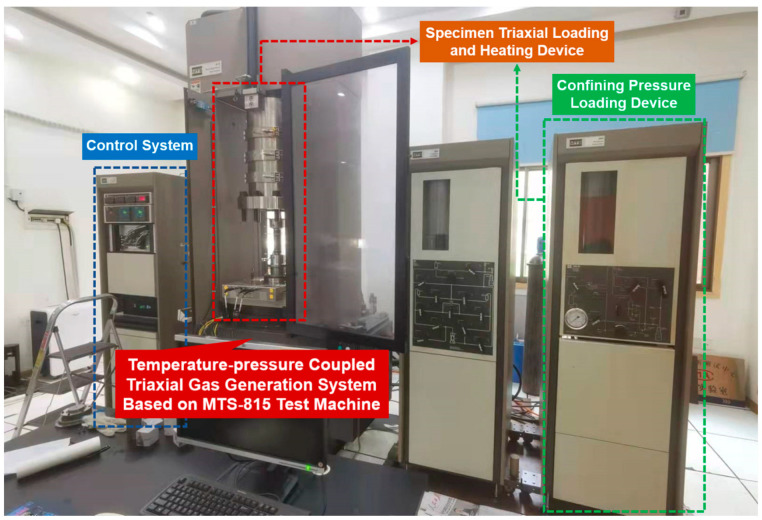
MTS-815 triaxial loading test system.

As illustrated in Figure 2, the temperature–pressure-coupled triaxial gas generation device and the confining pressure loading device in the laboratory are relatively independent of each other, and a single test condition can be changed in the test to simulate the geological environment of coal-rock at different burial depths and different temperatures. The triple-sealing measures of a heat-shrinkable sleeve, a self-melting adhesive, and reinforced steel wire can ensure good air tightness of the device and provide the premise for the accuracy of the test results.

#### 3.1.1. Temperature–Pressure-Coupled Triaxial Gas Generation Device

**Figure 3:**
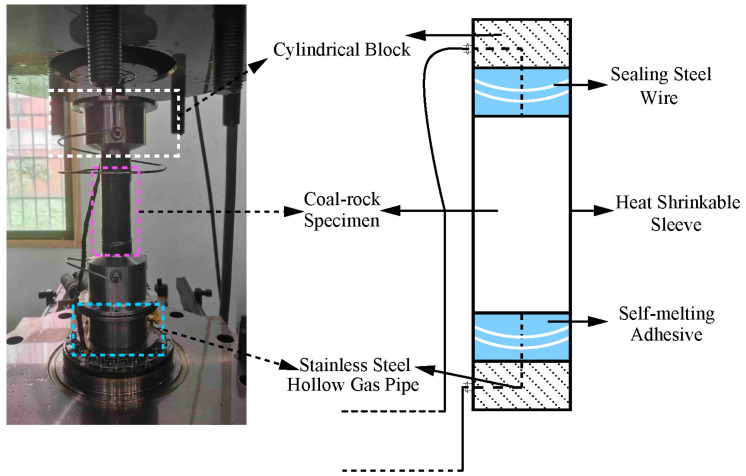
The specimen components.

The temperature–pressure-coupled triaxial gas generation device is mainly composed of specimen components, a triaxial chamber, an axial load device, and a heating device. As illustrated in Figure 3, the specimen components are mainly composed of two cylindrical blocks at the end, a stainless-steel hollow gas pipe, and the coal-rock specimen. First, the coal-rock specimen is fixed in the middle of the two end cylindrical blocks with the heat-shrinkable sleeve, and the heat-shrinkable sleeve is closely attached to the specimen with heating equipment such as a hot air blower. Then, the end blocks are sealed and connected to the heat-shrinkable sleeve with the self-melting adhesive. Finally, the sealing steel wire is wound for more than three turns to ensure that the air pressure will not tear the bonding of the self-melting adhesive during the test and prevent the high-temperature silicone oil in the triaxial chamber from infiltrating the heat-shrinkable sleeve and making contact with the coal-rock specimen. L-shaped stainless-steel hollow air pipes in the two end blocks are used to transport the coal gas to the exhaust device.

**Figure 4:**
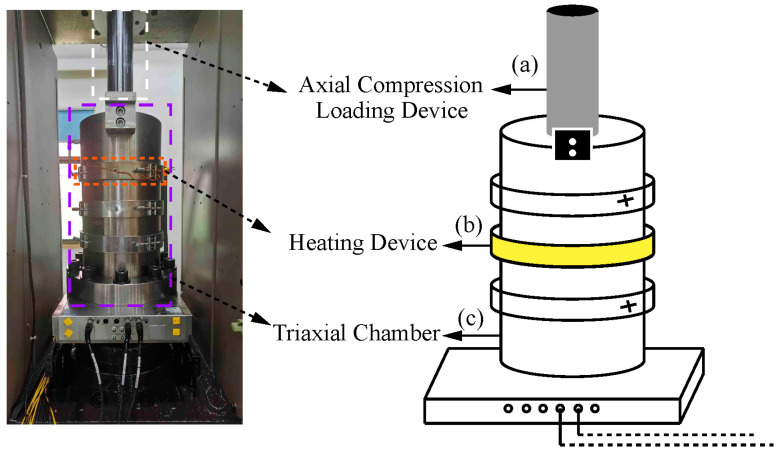
Triaxial chamber, axial load device, and heating device.

Figure 4 shows the other main components of the temperature–pressure-coupled triaxial gas generation device. (a) The axial load device is installed on the upper end of the box body of the triaxial chamber, including the loading frame, actuator, hydraulic power source, servo valve, and other components. During the test, the specimen components are axially compressed from top to bottom. (b) The heating device includes the heating strip wrapped around the outer wall of the triaxial chamber and the thermocouple temperature sensors placed on the pedestal of the triaxial chamber and close to the specimen components. In the test, the high-temperature silicone oil in the triaxial chamber is continuously heated from room temperature to 200 °C by the heating strip, and temperature feedback and control are carried out by the thermocouple temperature sensors. In the traditional coal-rock gas production test device, the temperature sensors are usually installed outside the cylinder block, which creates the problem of internal and external temperature differences. However, in this device, the temperature sensors are placed inside the triaxial chamber, close to the specimen components, and can directly measure the temperature of the environment where the specimen is located, avoiding the temperature difference between the inside and outside of the cylinder block and reducing the test error. (c) The triaxial chamber comprises a triaxial chamber box and a triaxial chamber pedestal, and the specimen components are placed inside. The triaxial chamber box is tubular, and the pedestal of the triaxial chamber is fixedly connected through a number of fastening bolts and fastening screws. The inlets and outlets of the high-temperature-resistant silicone oil connecting the oil inlet and outlet pipes in the confining pressure loading device are arranged on the box body and the box cover, respectively. During the test, the confining pressure loading device exerts triaxial compression on the specimen components by injecting silicone oil into the closed triaxial chamber.

#### 3.1.2. Confining Pressure Loading Device

The confining pressure loading device mainly includes a high-temperature silicone oil chamber, an inlet oil pipe and an outlet oil pipe connecting the oil chamber and the triaxial chamber, and a control structure driving the high-temperature-resistant silicone oil for circulating flow. The thermal decomposition temperature of the high-temperature-resistant silicone oil is higher than 300 °C, which is a suitable heat-transfer medium. It has good thermal stability and electrical insulation and is non-toxic and odorless.

### 3.2. Gas Collection and Monitoring Device

**Figure 5:**
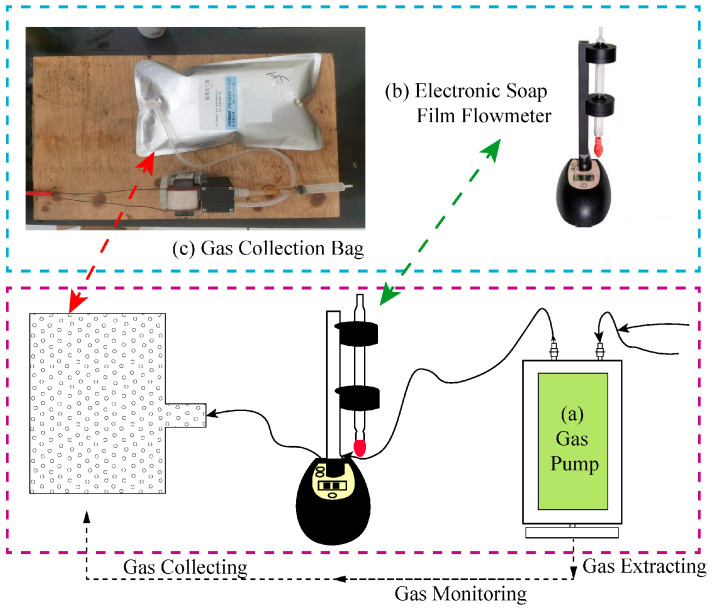
Gas collection and monitoring device.

The gas collection and monitoring device shown in Figure 5 mainly includes a gas extraction pump connected to the specimen components, an electronic soap film flowmeter, and a gas collection bag.

(a) Gas extraction pump. In previous coal-rock gas generation tests, there was generally no gas pump switch to control gas collection. The gas discharged from the specimen is often collected after the collection device is installed, which leads to the collection of the air in the test system before the start of the test and the omission of the residual gas in the system after the end of the test, thus affecting the accuracy of the experiment. However, in this study, the device is vacuumized by the gas extraction pump before the test, and all the residual gas in the test system is collected into the collection bag by the gas extraction pump after the test, which will greatly reduce the experimental error.

(b) High-precision electronic soap film flowmeter. This is connected between the gas extraction pump and the gas collection bag. The gases produced in the test are extracted by the gas pump and then pass through the electronic soap film flowmeter to measure the gas production rate and total gas yield of coal-rock under different conditions in real time.

(c) Gas collection bag. All the gases generated in the test are collected by the gas collection bag, which is more convenient and preserves the gas and analyzes it later more easily compared with the drainage method in the previous coal-rock gas generation tests.

### 3.3. Main Technical Parameters

For the developed gas production measurement system of coal-rock under temperature–pressure coupling, its main technical parameters are as follows: Load range: 0~2600 kN; load precision: ±0.5%; heating range: room temperature ~200 °C; confining pressure range: 0~140 MPa; flowmeter range: 0~100 mL·min^−1^.

## 4. Preliminary Test

In order to verify the functionality and practicability of the developed test system, representative coal-rock in China was selected for the temperature–pressure-coupled gas production test. The actual geological environment of coal-rock was simulated by applying axial pressure, confining pressure, and heat treatment on coal-rock specimens to explore the gas production mechanism of coal-rock under triaxial stress and geothermal conditions.

### 4.1. Specimen Preparation

Qinshui Basin in Shanxi Province is one of the coal-bearing basins with large coal reserves in China. The coal-bearing area is more than 40,000 square kilometers. The basin has a simple structure, stable coal seam occurrence, and large CBM reserves, which is a promising area for the exploitation of CBM resources in China.

In this test, in order to explore the gas composition and total gas yield generated by coal-rock deformation, anthracite with R_o, Max_ = 3.6% from the Shanxi Formation in Qinshui Basin was selected for the temperature–pressure-coupled deformation test in this paper. To reduce the anisotropy effect, all the tested specimens were cored from a single coal-rock block along the same direction, and then the obtained coal-rock specimens were processed into cylindrical specimens with the dimensions of φ50 mm × 100 mm, and the weight of each specimen was measured to be approximately 135g. The ends of each specimen were polished to be smooth and parallel in accordance with the ISRM standard [37].

### 4.2. Test Scheme

Since the pyrolysis reaction of coal-rock begins at 150 °C, in order to avoid the influence of coal pyrolysis on the experimental results and the slow reaction rate caused by the low temperature, the test temperature was set at 100 °C after comprehensive consideration. The confining pressure was set to 20 MPa, 30 MPa, and 40 MPa, and the axial pressure was set to 40 MPa based on the uniaxial compression test results, which was slightly higher than the confining pressure to simulate the stress environment of coal-rock at deep buried depths. At the same time, in order to avoid the premature failure of coal-rock specimens caused by excessive axial pressure or confining pressure, the axial pressure and confining pressure were increased synchronously in accordance with the corresponding rate during loading and were manually adjusted if necessary. After the above three conditions were set up, the temperature–pressure-coupled gas generation test was carried out on the coal-rock specimen.

Before the test, after the specimen components were installed on the test device, the triaxial chamber was put down and filled with silicone oil. First, vacuum treatment was performed for more than 30 min to remove the remaining gas in the coal-rock pores and the air in the test device. Then, the temperature of the silicone oil was heated to 100 °C and maintained. The gas desorbed by the coal-rock specimen at the temperature of 100 °C was collected by the gas collection device until the flowmeter monitoring showed that no gas was generated, and then the vacuum treatment was performed again. After loading the axial pressure and confining pressure to the design values, the gas production test of coal-rock was started. The test time was set to 72 h, and the gas generation was monitored by the high-precision flowmeter during the loading process. After reaching the desired time, the pressure relief and cooling treatment were performed on the test system, and the residual gas in the test device was extracted by the gas pump to ensure the accuracy of the test results. Finally, the gases collected in the two stages were analyzed by GC-MS to determine the composition and content of the collected gases.

### 4.3. Test Results

The gas production volume is one of the important parameters reflecting the gas production efficiency of coal-rock [29]. After 72 h of triaxial pressure–temperature-coupled tests, all gases were collected, and the concentration of methane was detected by GC-MS, as tabulated in Table 1. It can be seen that the total gas production volume collected from the coal-rock specimen under the confining pressure of 20 MPa, 30 MPa, and 40 MPa was 548 mL, 573 mL, and 607 mL, and the measured methane concentration was 4778.92 ppm, 4854.15 ppm, and 4897.63 ppm, so the total gas production volume of methane can be calculated by Equation (1) as 2.619 mL, 2.781 mL, and 2.973 mL, respectively.

(1) $$Q_{TM}=C_{M}Q_{T}\times 10^{-6}$$

where *Q_TM_* represents the total production volume of methane, mL; *C_M_* is the measured concentration of methane, ppm; and *Q_T_* is the total production volume of all gases collected in the test, mL.

The residual methane content of coal-rock specimens SX-20, SX-30, and SX-40 measured by the laboratory test was 0.015 mL/g, 0.013 mL/g, and 0.012 mL/g, respectively. Since the weight of the standard coal-rock specimen is 135 g, the volume of residual methane (*Q_RM_*) contained in the coal-rock specimen is 2.025 mL, 1.755 mL, and 1.620 mL, respectively. Then, the actual production volume of methane by coal-rock deformation (*Q_DM_*) under the temperature–pressure coupling can be calculated by Equation (2) as 0.594 mL, 1.026 mL, and 1.353 mL, respectively.

(2) $$Q_{DM}=Q_{TM}-Q_{RM}$$

From this, it is certain that methane and other gases were generated from the coal-rock specimen under the coupled effect of triaxial pressure and temperature. In addition, it could be found that with the increase in confining pressure, the total production volume of methane and other gases generated by coal-rock deformation both increased, and the concentration of methane also increased slightly.

The composition analysis by GC-MS showed that CO was the main gas, except methane, in the triaxial pressure–temperature-coupled test. The mass fraction of carbon, hydrogen, and other elements in the coal-rock specimen also decreased, indicating that the gas generated by coal-rock deformation may mainly come from the change in the chemical structure of the coal-rock itself [29,30].

## 5. Conclusions

(1) Considering the sub-high-temperature and sub-high-pressure environments and long-term loading requirements for gas production by coal-rock deformation, a triaxial gas production measurement system of coal-rock under temperature–pressure coupling was developed. The system has the functions of axial loading, confining pressure loading, continuous heating, gas collection, etc., and has the characteristics of high reliability, good air tightness, and long-term loading and heating.

(2) The developed test system was used to conduct a preliminary test on the gas production of anthracite under temperature–pressure coupling. It was found that the gas yield generated by coal-rock deformation increased with increasing confining pressure. In addition, GC-MS analysis showed that the main components of the gas generated by coal-rock deformation included CO, methane, and other hydrocarbon gases, which verified the generation of methane in the anthracite deformation.

(3) The functionality and practicability of the developed test system were verified by the preliminary test. However, only the change in confining pressure was considered in the present study. Therefore, more systematic gas production tests will be carried out on different coal-rocks under different temperatures and triaxial pressures, so as to deepen the understanding of the gas production mechanism in coal-rock deformation.

## Figures and Tables

**Table 1:** Total production volume of gas and methane collected in the test.

Specimen No.	*T*/°C	*σ*_1_/MPa	*σ*_3_/MPa	*Q_T_*/mL	*C_M_*/ppm	*Q_DM_*/mL
SX-20	100	40	20	548	4778.92	0.594
SX-30	100	40	30	573	4854.15	1.026
SX-40	100	40	40	607	4897.63	1.353

## Data Availability

Not applicable.
